# Consensus-Based Filter for Distributed Sensor Networks with Colored Measurement Noise

**DOI:** 10.3390/s18113678

**Published:** 2018-10-29

**Authors:** Jinran Wang, Peng Dong, Zhongliang Jing, Jin Cheng

**Affiliations:** 1 School of Aeronautics and Astronautics, Shanghai Jiao Tong University, Shanghai 200240, China; wangelmo@sjtu.edu.cn; 2 Science and Technology on Complex System Control and Intelligent Agent Cooperation Laboratory, Beijing Electro-Mechanical Engineering Institute, Beijing 100074, China; 13301102393@189.cn

**Keywords:** colored noise, consensus filter, distributed state estimation, distributed sensor network

## Abstract

Consensus filtering is an effective method for distributed state estimation of distributed sensor networks and the assumption of white measurement noise is widely used. However, when the measurement noise is colored, the traditional consensus filter cannot work well. In this paper, we first propose a consensus-based distributed filter for colored measurement noise by augmenting the state to include the colored measurement noise. To improve the efficiency of the filter, only local colored measurement noise is integrated into the augmented state for each local filter. Furthermore, another consensus-based distributed filter based on measurement differencing scheme is developed to eliminate the ill-conditioned computations of the augmented state approach. In addition, this method does not need to augment the state and thus has lower dimension than the augmented state filter. Simulation results demonstrate the superiority of the proposed methods.

## 1. Introduction

Distributed state estimation has drawn a lot of attention in recent years for its advantages. There is no need for the fusion center to gather raw measurements of all nodes in the sensor network [1]. Therefore, it is more robust to the failure of filtering or communication than the centralized one [2,3]. In addition, distributed state estimation can achieve higher scalability to the changes in the sensor network since it dose not need the global topology knowledge of the whole sensor network [1,4].

There are many strategies to process distributed state estimation. One of these strategies is the sequential method [5]. In this scheme, nodes are successively selected and information are transmitted and processed sequentially in the sensor network. Another strategy is the iterative scheme where each node exchanges its local information with its neighbor nodes iteratively. It contains consensus algorithms [6,7,8,9,10], gossip methods [11,12,13] and diffusion strategies [14,15,16]. Among them, consensus method is an effective tool for distributed state estimation of sensor network to achieve global consistency of all nodes. Each node communicates with its neighbors to cooperatively compute a sum, maximum, or average according to an iterative way in consensus-based filters. Moreover, they are more robust to node or link failures. The quantities that can be exchanged between neighboring nodes are local estimate [17], local posteriors, local measurements [18], and information quantities [19]. In recent years, many extension methods [20,21,22,23,24,25,26] have been proposed based on these basic approaches.

The measurement noise characteristics play an important role in state estimation problem. However, the aforementioned distributed state estimation methods assume that the measurement noise is white. This assumption is too restrictive, and applications with non-white noise frequently arise in practice such as speech signal processing, radar signal processing and signal processing of Global Navigation Satellite System (GNSS) [27,28,29]. Thus, when the measurement noise is colored, these filters cannot work well. The colored noise is often described by a linear system model with white noise, and many methods are proposed to deal with colored noise based on Kalman filter. The most direct way is to augment the state to include the time correlated colored measurement noise [30,31]. The state augmentation approach is also used in the colorfulness of speech signal [32]. For this augmented system, the measurements contain no noise, which may cause the problem of ill-conditioned computations in constructing the data processing filter. To deal with the singular problem, another filtering method based on measurement differencing is proposed in [33,34]. It is effective to remove the time-correlated part of the measurement noise, which makes it capable of converting the received colored noise into white noise [35]. The measurement differencing method has also been used in nonlinear systems with colored measurement noise to tackle the state filtering and smoothing problem based on Gaussian approximation [36]. In addition, it is extended to multiplicative noises in [37,38] and robust state estimation for centralized fusion in [39]. However, the above methods about colored noise do not take distributed state estimation for sensor network into consideration.

In this paper, we focus on the distributed state estimation problem for sensor network with colored measurement noise which is the output of a discrete-time linear system with white noise. The consensus distributed filter for colored measurement noise based on state augmentation is derived first. Since the augmentation of colored measurement noise fo all sensor nodes may cause high-dimensional matrix calculation, we only augment the local colored measurement noise into the local state. Moreover, only the state and error covariance without local colored measurement noise are exchanged with neighboring nodes and achieved consensus. In the state augmentation method, the correlation matrix of the measurement noise is singular for there is no noise in the measurements. To eliminate the ill-conditioned computations of the augmented state approach and reduce the dimension of the augmentation state, the consensus-based distributed filter based on measurement differencing is proposed. Simulations are processed for sensor network with colored measurement noises. Results show that the proposed methods outperform the traditional consensus-based distributed state estimation method.

The reminder of this paper is organized as follows. Section 2 describes models of sensor network and gives the problem formulation. Section 3 presents our consensus-based distributed state filters for sensor network with colored measurement noise. Numerical results and analysis are given in Section 4 and the conclusion is given in Section 5.

## 2. Background

### 2.1. Problem Formulation

This paper considers a sensor network denoted by (N,A), where N is the set of sensor nodes and A⊆N×N denotes the set of connections between nodes such that (i,j)∈A if node *j* can receive data from the node *i*. In addition, for any node i∈N, we use a set $N^{i}$ that represents all of the node’s neighbors (including *i* itself), i.e., $N^{i}\overset{\Delta }{\;=}\left\{j:\left(j,i\right)\in A\right\}$.

Consider a discrete-time linear stochastic system

(1) $$x_{k}=F_{k-1}x_{k-1}+w_{k-1}$$

and measurement equations of the sensor node i∈N

(2) $$z_{k}^{i}=H_{k}^{i}x_{k}+v_{k}^{i},\;i\in N$$

where $x_{k}$ is the *n*-dimension state vector, $F_{k}$ is the state transition matrix, $w_{k}$ is the process noise, $z_{k}^{i}$ is the *d*-dimension measurement vector of the sensor node i∈N, $H_{k}^{i}$ is the measurement matrix of the sensor node i∈N, $v_{k}^{i}$ is the measurement noise of each sensor node i∈N.

Then some assumptions are given to approximate conditional distributions and dynamic of these parameters. For the process noise $w_{k}$ and measurement noise $v_{k}^{i}$, we have the following assumptions.

**Assumption** **1.** *The process noise $w_{k}$ is white Gaussian of which the statistical character is given as*

(3) $$w_{k}∼N\left(0,Q_{k}\right)$$

(4) $$E\left[w_{k}w_{j}^{T}\right]=Q_{k}\delta_{k-j}$$

**Assumption** **2.** *The measurement noise $v_{k}^{i}$ is colored noise which is correlated with itself at different time stamps and the model of measurement noise is given as*

(5) $$v_{k}^{i}=\Psi_{k-1}^{i}v_{k-1}^{i}+\xi_{k}^{i},\;i\in N$$

*where $\Psi_{k-1}^{i}$ is noise transition matrix, $\xi_{k}^{i}$ is zero mean white noise which is uncorrelated with $v_{k-1}$ and the statistical character of each noise is given by*

(6) $$\xi_{k}^{i}∼N\left(0,R_{k}^{i}\right)$$

(7) $$E\left[\xi_{k}^{i}{\left(\xi_{j}^{i}\right)}^{T}\right]=R_{k}^{i}\delta_{k-j}$$

(8) $$E[v_{k}v_{k-i}^{T}]\neq 0,\;i>0$$

**Assumption** **3.** *The process noise and the measurement noise are uncorrelated*

(9) $$E[w_{k}{\left(\xi_{j}^{i}\right)}^{T}]=0$$

**Figure 1:**
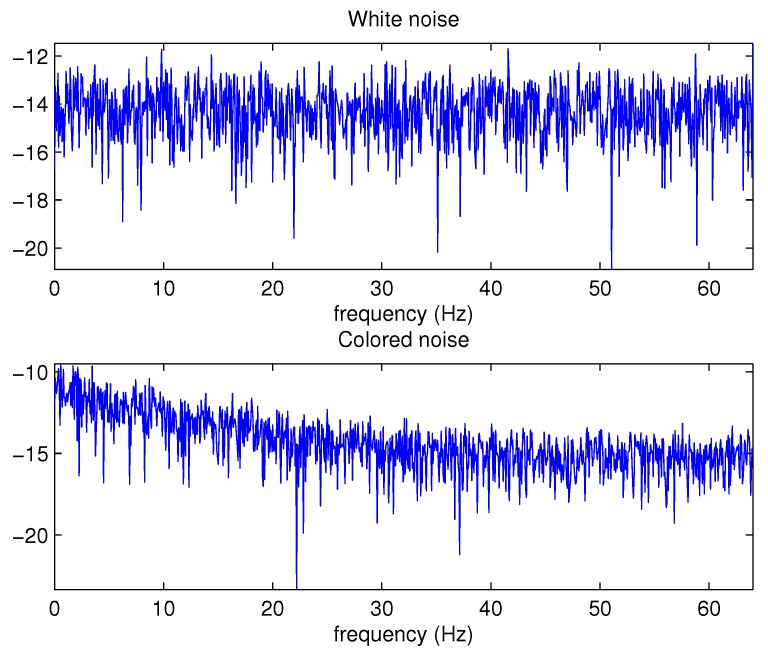
Power spectral densities of white noise and colored noise.

Here we give an example figure of comparison between white noise and colored noise. Figure 1 shows that power spectral density of white noise distributes almost uniformly throughout the frequency domain while the power spectral density of colored noise decreases with frequency.

The purpose of consensus filter in this paper is to obtain a consensus state of a sensor network with colored measurement noise.

### 2.2. Consensus Algorithm

Consensus algorithm is the information exchange rule that ensures that the amount of concern of each node achieves consistency. We define the weighted Kullback-Leibler average (KLA) $\bar{p}\left(\cdot \right)$ among the PDFs $\left\{p^{i}\left(\cdot \right)\right\}$ according to [1]

(10) $$\bar{p}=\arg\underset{p(\cdot )}{\inf}\sum_{i\in N}\pi^{i}KL(p||p^{i})$$

where $\pi^{i}>0$ is the weight satisfying $\sum_{i\in N}\pi^{i}=1$, $KL(p||p^{i})$ is the Kullback-Leibler divergence (KLD) between the PDFs p(·) and $p^{i}\left(\cdot \right)$. The probability density consensus problem can be described as finding a consensus algorithm to make

(11) $$\lim_{l\to +\infty }p_{l}^{i}=\bar{p}\left(x\right),\forall i\in N$$

where $\bar{p}\left(\cdot \right)$ is the asymptotic PDF. Then the solution to (10) is

(12) $$\bar{p}\left(x\right)=\frac{\prod_{i\in N}{\left[p^{i}\left(x\right)\right]}^{\pi^{i}}}{\int \prod_{i\in N}{\left[p^{i}\left(x\right)\right]}^{\pi^{i}}dx}\overset{\Delta }{\;=}\underset{i\in N}{⨁}\left(\pi^{i}⊙p^{i}\left(x\right)\right)$$

where $\pi^{i}=1/\left|N\right|$, the operators ⊕ and ⊙ are defined by

(13) $$p\left(x\right)⊕q\left(x\right)\overset{\Delta }{\;=}\frac{p(x)q(x)}{\int p(x)q(x)dx}$$

(14) $$\pi ⊙p\left(x\right)\overset{\Delta }{\;=}\frac{{\left[p\left(x\right)\right]}^{\pi }}{\int {\left[p\left(x\right)\right]}^{\pi }dx}$$

Therefore, the solution can be obtained by exchanging the local data with the neighbors via convex combination in a iterative way

(15) $$p_{\ell }^{i}\left(x\right)=\underset{j\in N^{i}}{⨁}\left(\pi^{i,j}⊙p_{\ell -1}^{j}\left(x\right)\right)$$

where $\pi^{i,j}\geq 0$ is the consensus weight with $\sum_{j\in N^{i}}\pi^{i,j}=1$, *ℓ* is the iteration index and iterations are initialized with $p_{0}^{i}\left(x\right)=p^{i}\left(x\right)$.

If the PDFs are Gaussian such as $p^{i}\left(x\right)=N\left(x^{i},P^{i}\right)$, the average PDF can be obtained by averaging information matrices $\Omega^{i}={\left(P^{i}\right)}^{-1}$ and information vectors $q^{i}=\Omega^{i}{\hat{x}}^{i}$. Given the information matrices and the information vectors, we have the following consensus algorithm

(16) $$\Omega_{\ell }^{i}=\sum_{j\in N^{i}}\pi^{i,j}\Omega_{k,\ell -1}^{j}$$

(17) $$q_{\ell }^{i}=\sum_{j\in N^{i}}\pi^{i,j}q_{\ell -1}^{j}$$

**Remark** **1.** *It should be noted that the consensus is only achieved when the network is strongly connected. In the network S=(N,A), if there are paths from a to b and from b to a for any two different vertices a and b in N, then S is said to be a strongly connected network.*

## 3. Proposed Method

### 3.1. Augmented State Approach

The direct way to solve the colored measurement noise problem is augmented state approach. This augmented state approach is originally proposed in [30] for single sensor. We will extend it to multiple sensors in a distributed way in the following part.

We augment the original system model for multiple sensors as follows:

(18) $$\left[\begin{array}{c} x_{k}\\ v_{k} \end{array}\right]=\left[\begin{array}{cc} F_{k-1} & 0\\ 0 & \Psi_{k-1} \end{array}\right]\left[\begin{array}{c} x_{k-1}\\ v_{k-1} \end{array}\right]+\left[\begin{array}{c} w_{k-1}\\ \xi_{k-1} \end{array}\right]$$

where

$$v_{k}={\left[{\left(v_{k}^{1}\right)}^{T},{\left(v_{k}^{2}\right)}^{T},\ldots ,{\left(v_{k}^{i}\right)}^{T},\ldots \right]}^{T},\;i\in N$$

$$\xi_{k}={\left[{\left(\xi_{k}^{1}\right)}^{T},{\left(\xi_{k}^{2}\right)}^{T},\ldots ,{\left(\xi_{k}^{i}\right)}^{T},\ldots \right]}^{T},\;i\in N$$

$$\Psi_{k}=diag\left[\Psi_{k}^{1},\Psi_{k}^{2},\ldots ,\Psi_{k}^{i},\ldots \right],\;i\in N$$

Since the measurement noise among each sensor node is independent, then the measurement model for each sensor node i∈N becomes

(19) $$z_{k}^{i}=\left[\begin{array}{cc} H_{k}^{i} & I \end{array}\right]\left[\begin{array}{c} x_{k}\\ v_{k}^{i} \end{array}\right]+0,\;i\in N$$

If we run consensus on the augmented state, we will not only exchange the original state but also the noise states of all sensors with neighbors. However, the communication capability of sensor network may be too limited to afford these large data. Moreover, the quantity we interested in sensor network is the original state instead of the augmented state. Thus, we suggest that the consensus is carried out only on the original state and each sensor node runs a local filter with a local augmented state. Then the system model for each local sensor node i∈N becomes

(20) $$\left[\begin{array}{c} x_{k}\\ v_{k}^{i} \end{array}\right]=\left[\begin{array}{cc} F_{k-1} & 0\\ 0 & \Psi_{k-1}^{i} \end{array}\right]\left[\begin{array}{c} x_{k-1}\\ v_{k-1}^{i} \end{array}\right]+\left[\begin{array}{c} w_{k-1}\\ \xi_{k-1}^{i} \end{array}\right]$$

The system model (20) and measurement model (19) can be rewritten as

(21) $${x^{\prime }}_{k}^{i}={F^{\prime }}_{k-1}^{i}{x^{\prime }}_{k-1}^{i}+{w^{\prime }}_{k-1}^{i},\;i\in N$$

(22) $$z_{k}^{i}={H^{\prime }}_{k}^{i}{x^{\prime }}_{k}^{i}+{v^{\prime }}_{k}^{i},\;i\in N$$

where ${v^{\prime }}_{k}^{i}=0$ and

$${x^{\prime }}_{k}^{i}=\left[\begin{array}{c} x_{k}\\ v_{k}^{i} \end{array}\right]$$

$${F^{\prime }}_{k}^{i}=\left[\begin{array}{cc} F_{k} & 0\\ 0 & \Psi_{k}^{i} \end{array}\right]$$

$${w^{\prime }}_{k}^{i}=\left[\begin{array}{c} w_{k}\\ \xi_{k}^{i} \end{array}\right]$$

$${H^{\prime }}_{k}^{i}=\left[\begin{array}{cc} H_{k}^{i} & I \end{array}\right]$$

Then the covariance of the process noise and the covariance of the measurement noise can be computed as

(23) $$\begin{array}{cc} {Q^{\prime }}_{k}=E\left[{w^{\prime }}_{k}^{i}{\left({w^{\prime }}_{k}^{i}\right)}^{T}\right] & =E\left[\begin{array}{cc} \left(\begin{array}{c} w_{k}\\ \xi_{k}^{i} \end{array}\right) & \left(\begin{array}{cc} w_{k}^{T} & {\left(\xi_{k}^{i}\right)}^{T} \end{array}\right) \end{array}\right]\\ & =\left[\begin{array}{cc} Q_{k} & 0\\ 0 & R_{k}^{i} \end{array}\right] \end{array}$$

(24) $$E[{v^{\prime }}_{k}^{i}{\left({v^{\prime }}_{k}^{i}\right)}^{T}]=0$$

Using the modified system model (21) and measurement model (22) together with the consensus algorithm in Section 2.2, we can obtain the consensus filter for colored measurement noise by augmented state approach.

Suppose we have the augmented state estimate $\hat{x}{{}^{\prime }}_{k-1}^{i}$ and the corresponding state error covariance ${P^{\prime }}_{k-1}^{i}$ for each node i∈N, then we can proceed the distributed recursion of consensus filter at time *k* as follows.

(1) Prediction of local filter: Compute the predicted augmented state estimate $\hat{x}{{}^{\prime }}_{k|k-1}^{i}$ and the corresponding state error covariance ${P^{\prime }}_{k|k-1}^{i}$
  (25) $$\hat{x}{{}^{\prime }}_{k|k-1}^{i}={F^{\prime }}_{k-1}^{i}\hat{x}{{}^{\prime }}_{k-1}^{i}$$
  (26) $$P{{}^{\prime }}_{k|k-1}^{i}=F{{}^{\prime }}_{k-1}^{i}P{{}^{\prime }}_{k-1}^{i}{\left(F{{}^{\prime }}_{k-1}^{i}\right)}^{T}+Q{{}^{\prime }}_{k-1}$$
(2) Update of local filter: Calculate the residual and the associated covariance
  (27) $${\tilde{z}}_{k}^{i}=z_{k}^{i}-H{{}^{\prime }}_{k}^{i}\hat{x}{{}^{\prime }}_{k|k-1}^{i}$$
  (28) $${S^{\prime }}_{k}^{i}=H{{}^{\prime }}_{k}^{i}P{{}^{\prime }}_{k|k-1}^{i}{\left(H{{}^{\prime }}_{k}^{i}\right)}^{T}$$
  Calculate the local filter gain
  (29) $$K{{}^{\prime }}_{k}^{i}=P{{}^{\prime }}_{k|k-1}^{i}{\left(H{{}^{\prime }}_{k}^{i}\right)}^{T}/S{{}^{\prime }}_{k}^{i}$$
  Update the augmented state and the associated error covariance
  (30) $$\hat{x}{{}^{\prime }}_{k,0}^{i}=\hat{x}{{}^{\prime }}_{k|k-1}^{i}+K{{}^{\prime }}_{k}^{i}{\tilde{z}}_{k}^{i}$$
  (31) $$P{{}^{\prime }}_{k,0}^{i}=P{{}^{\prime }}_{k|k-1}^{i}-K{{}^{\prime }}_{k}^{i}S{{}^{\prime }}_{k}^{i}{\left(K{{}^{\prime }}_{k}^{i}\right)}^{T}$$
(3) Consensus on the original information matrices and information vectors: For a *L*-step consensus iteration, the consensus on posterior information is carried out in the form
  (32) $$\Omega_{k,\ell }^{i}=\sum_{j\in N^{i}}\pi^{i,j}\Omega_{k,\ell -1}^{j},\;\ell =1,\ldots ,L$$
  (33) $$q_{k,\ell }^{i}=\sum_{j\in N^{i}}\pi^{i,j}q_{k,\ell -1}^{j},\;\ell =1,\ldots ,L$$
  (34) $$P_{k}^{i}={\left(\Omega_{k,L}^{i}\right)}^{-1}$$
  (35) $${\hat{x}}_{k}^{i}=P_{k}^{i}q_{k,L}^{i}$$
  where ℓ=1,2,⋯,L is the consensus step, $\pi^{i,j}$ is the consensus weight. A convex combination is adopted by supposing $\pi^{i,j}\geq 0$ and $\sum_{j\in N^{i}}\pi^{i,j}=1$ [8]. The iteration is initialized by
  (36) $$\Omega_{k,0}^{i}={\left(P_{k,0}^{i}\right)}^{-1}$$
  (37) $$q_{k,0}^{i}=\Omega_{k,0}^{i}{\hat{x}}_{k,0}^{i}$$
  where $P_{k,0}^{i}=P{{}^{\prime }}_{k,0}^{i}\left(1:n,1:n\right)$, ${\hat{x}}_{k,0}^{i}=\hat{x}{{}^{\prime }}_{k,0}^{i}\left(1:n\right)$ and *n* is the dimension of the original state.
(4) Reset the local filter:
  (38) $$P{{}^{\prime }}_{k}^{i}=\left[\begin{array}{cc} P_{k}^{i} & 0\\ 0 & P_{v_{k}^{i}} \end{array}\right]$$
  $$\hat{x}{{}^{\prime }}_{k}^{i}=\left[\begin{array}{c} {\hat{x}}_{k}^{i}\\ {\hat{v}}_{k}^{i} \end{array}\right]$$
  where $P_{v_{k}}^{i}={P^{\prime }}_{k}^{i}\left(n+1:n+d,n+1:n+d\right)$, ${\hat{v}}_{k}^{i}=\hat{x}{{}^{\prime }}_{k}^{i}\left(n+1:n+d\right)$ and *d* is the dimension of the measurement of each node.

### 3.2. Measurement Differencing Approach

However, when constructing a data processing filter, the augmented state process may result in ill-conditioned calculations. The absence of noise in some measurements is a singular problem because in the Kalman filtering, the correlation matrix of the measured noise is singular, i.e., there is no *R*. Instead, another filter capable of converting the received colored noise into white noise called measurement differencing is proposed for single sensor in [33]. Similarly, we present a measurement differencing-based distributed filter for sensor networks in this section. The contribution of the colored portion of the noise to the received signal is first estimated and then subtracted from the received signal in each sensor node. This method has a lower dimensionality than the augmented state method.

For each sensor node, we define an auxiliary measurement signal:

(39) $${z^{\prime }}_{k-1}^{i}=z_{k}^{i}-\Psi_{k-1}^{i}z_{k-1}^{i},\;i\in N$$

Substituting $z_{k}^{i}$ and $z_{k-1}^{i}$ into (39), we can obtain

(40) $$\begin{array}{cc} {z^{\prime }}_{k-1}^{i} & =H_{k}^{i}x_{k}+v_{k}^{i}-\Psi_{k-1}^{i}\left(H_{k-1}^{i}x_{k-1}+v_{k-1}^{i}\right)\\ & =H_{k}^{i}\left(F_{k-1}x_{k-1}+w_{k-1}\right)+v_{k}^{i}-\Psi_{k-1}^{i}\left(H_{k-1}^{i}x_{k-1}+v_{k-1}^{i}\right)\\ & =\left(H_{k}^{i}F_{k-1}-\Psi_{k-1}^{i}H_{k-1}^{i}\right)x_{k-1}+H_{k}^{i}w_{k-1}+v_{k}^{i}-\Psi_{k-1}^{i}v_{k-1}^{i}\\ & =\left(H_{k}^{i}F_{k-1}-\Psi_{k-1}^{i}H_{k-1}^{i}\right)x_{k-1}+H_{k}^{i}w_{k-1}+\xi_{k-1}^{i}\\ & =H_{k-1}^{{}^{\prime }i}x_{k-1}+v_{k-1}^{{}^{\prime }i} \end{array}$$

where

$$H{{}^{\prime }}_{k-1}^{i}=H_{k}^{i}F_{k-1}-\Psi_{k-1}^{i}H_{k-1}^{i}$$

$${v^{\prime }}_{k-1}^{i}=H_{k}^{i}w_{k-1}+\xi_{k-1}^{i}$$

Thus, we obtain a new measurement equation where the measurement is ${z^{\prime }}_{k-1}^{i}$, the measurement matrix is $H{{}^{\prime }}_{k-1}^{i}$ and the measurement noise is ${v^{\prime }}_{k-1}^{i}$. Then we have a new but equivalent system for the sensor network:

(41) $$\begin{array}{cc} x_{k} & =F_{k-1}x_{k-1}+w_{k-1}\\ {z^{\prime }}_{k}^{i} & =H{{}^{\prime }}_{k}^{i}{x^{\prime }}_{k}^{i}+{v^{\prime }}_{k}^{i},\;i\in N \end{array}$$

where the time-correlated colored noise $v_{k}^{i}$ does not appear in the new measurement ${z^{\prime }}_{k}^{i}$. In addition, the measurement noise ${v^{\prime }}_{k}^{i}$ in ${z^{\prime }}_{k}^{i}$ is a zero-mean white Gaussian noise. Therefore, the covariance of the new measurement noise $v^{\prime }$, and the cross covariance between the process noise *w* and the new measurement noise $v^{\prime }$ can be obtained by

(42) $$\begin{array}{cc} E[{v^{\prime }}_{k}^{i}{\left({v^{\prime }}_{k}^{i}\right)}^{T}] & =E[\left(H_{k+1}^{i}w_{k}+\xi_{k}^{i}\right){\left(H_{k+1}^{i}w_{k}+\xi_{k}^{i}\right)}^{T}]\\ & =H_{k+1}^{i}Q_{k}{\left(H_{k+1}^{i}\right)}^{T}+R_{k}^{i} \end{array}$$

(43) $$\begin{array}{cc} E[w_{k}{\left({v^{\prime }}_{k}^{i}\right)}^{T}] & =E[w_{k}{\left(H_{k+1}^{i}w_{k}+\xi_{k}^{i}\right)}^{T}]\\ & =Q_{k}{\left(H_{k+1}^{i}\right)}^{T} \end{array}$$

We use the following notation to denote the actual estimates for each sensor node *i*: ${\hat{x}}_{j|k}^{i}=$ optimal estimate of $x_{j}$ given measurements up to and including $z_{k}^{i}$. That is

(44) $${\hat{x}}_{j|k}^{i}=E\left[x_{j}|Z_{k}^{i}\right]$$

where $Z_{k}^{i}=\left\{z_{1}^{i},z_{2}^{i},\ldots ,z_{k}^{i},i\in N\right\}$ denote measurements of the node *i* till time *k*. The error covariance of ${\hat{x}}_{j|k}^{i}$ is defined as

(45) $$P_{j|k}^{i}=E\left[\left(x_{j}-{\hat{x}}_{j|k}^{i}\right){\left(x_{j}-{\hat{x}}_{j|k}^{i}\right)}^{T}\right]$$

In the following part, we use the formal notion ${\bar{x}}_{k}^{i}={\hat{x}}_{k|k}^{i}$ and ${\hat{x}}_{k-1}^{i}={\hat{x}}_{k-1|k}^{i}$ to denote the formal prediction ${\bar{x}}_{k}^{i}$ and estimate ${\hat{x}}_{k-1}^{i}$ based on ${z^{\prime }}_{k-1}^{i}$, respectively. Moreover, the notion $M_{k}^{i}$ and $P_{k-1}^{i}$ is used to denote the error covariance of ${\bar{x}}_{k}^{i}$ and ${\hat{x}}_{k-1}^{i}$, respectively. The state estimate ${\hat{x}}_{k}^{i}$ at time *k* is defined as the expected value of the state $x_{k}$ conditioned on measurements of node *i* up to and including time k+1. We suppose that the state estimate ${\hat{x}}_{k}^{i}$ can be given by a standard linear combination as follows

(46) $${\hat{x}}_{k}^{i}={\bar{x}}_{k-1}^{i}-K_{k}^{i}\left({z^{\prime }}_{k}^{i}-{H^{\prime }}_{k}^{i}{\bar{x}}_{k-1}^{i}\right)$$

The gain $K_{k}^{i}$ can be obtained by minimizing the trace of the covariance of the estimation error. That is

(47) $$K_{k}^{i}=\arg\min\mathrm{Tr}E\left[\left(x_{k}-{\hat{x}}_{k}^{i}\right){\left(x_{k}-{\hat{x}}_{k}^{i}\right)}^{T}\right]$$

where Tr denotes the trace. We can use the filtering solution proposed in [33] to exactly compute ${\hat{x}}_{k}^{i}$ and $K_{k}^{i}$. The details of this minimization can be seen in [33,34,35]. Then we can obtain the distributed estimator for colored measurement noise as follows.

Suppose we have the predicted state estimate ${\bar{x}}_{k}^{i}$ and the corresponding state error covariance $M_{k}^{i}$ for each node i∈N, then we can proceed the distributed recursion of consensus filter at time *k* as follows.

(1) Update of local filter:
  (48) $${\hat{x}}_{k,0}^{i}={\bar{x}}_{k}^{i}+K_{k}^{i}{\tilde{z}}_{k}^{i}$$
  (49) $$P_{k,0}^{i}=M_{k}^{i}-K_{k}^{i}S_{k}^{i}{\left(K_{k}^{i}\right)}^{T}$$
  where
  (50) $${\tilde{z}}_{k}^{i}={z^{\prime }}_{k}^{i}-{H^{\prime }}_{k}^{i}{\bar{x}}_{k}^{i}$$
  (51) $${z^{\prime }}_{k}^{i}=z_{k+1}^{i}-\Psi_{k}^{i}z_{k}^{i}$$
  (52) $${H^{\prime }}_{k}^{i}=H_{k+1}^{i}F_{k}-\Psi_{k}^{i}H_{k}^{i}$$
  (53) $$K_{k}^{i}=M_{k}^{i}{\left({H^{\prime }}_{k}^{i}\right)}^{T}/S_{k}^{i}$$
  (54) $$S_{k}^{i}={H^{\prime }}_{k}^{i}M_{k}^{i}{\left({H^{\prime }}_{k}^{i}\right)}^{T}+{R^{\prime }}_{k}^{i}$$
  (55) $${R^{\prime }}_{k}^{i}=H_{k+1}^{i}Q_{k}{\left(H_{k+1}^{i}\right)}^{T}+R_{k}^{i}$$
(2) Consensus on the information matrices and information vectors: For a *L*-step consensus iteration, the consensus on posterior information is carried out by
  (56) $$\Omega_{k,\ell }^{i}=\sum_{j\in N^{i}}\pi^{i,j}\Omega_{k,\ell -1}^{j},\;\ell =1,\ldots ,L$$
  (57) $$q_{k,\ell }^{i}=\sum_{j\in N^{i}}\pi^{i,j}q_{k,\ell -1}^{j},\;\ell =1,\ldots ,L$$
  (58) $$P_{k}^{i}={\left(\Omega_{k,L}^{i}\right)}^{-1}$$
  (59) $${\hat{x}}_{k}^{i}=P_{k}^{i}q_{k,L}^{i}$$
  where ℓ=1,2,⋯,L is the consensus step, $\pi^{i,j}$ is the consensus weight. A convex combination is adopted by supposing $\pi^{i,j}\geq 0$ and $\sum_{j\in N^{i}}\pi^{i,j}=1$. The iteration can be initialized by
  (60) $$\Omega_{k,0}^{i}={\left(P_{k,0}^{i}\right)}^{-1}$$
  (61) $$q_{k,0}^{i}=\Omega_{k,0}^{i}{\hat{x}}_{k,0}^{i}$$
(3) Prediction of local filter:
  (62) $${\bar{x}}_{k+1}^{i}={\hat{x}}_{k}^{i}+D_{k}^{i}\left({z^{\prime }}_{k}^{i}-{H^{\prime }}_{k}^{i}{\hat{x}}_{k}^{i}\right)$$
  (63) $$M_{k}^{i}=\left(F_{k}-D_{k}^{i}{H^{\prime }}_{k}^{i}\right)P_{k}^{i}{\left(F_{k}-D_{k}^{i}{H^{\prime }}_{k}^{i}\right)}^{T}+Q_{k}-D_{k}^{i}{R^{\prime }}_{k}^{i}{\left(D_{k}^{i}\right)}^{T}$$
  where
  (64) $$D_{k}^{i}=C_{k}^{i}/S_{k}^{i}$$
  (65) $$C_{k}^{i}=Q_{k}{\left(H_{k+1}^{i}\right)}^{T}$$

It should be noted that when the first measurement $z_{1}^{i}$ for each node *i* comes, there is not yet sufficient information to calculate ${z^{\prime }}_{1}^{i}$. Therefore, the augmented state method should be used to obtain the optimal estimate of $x_{1}$ and the associated covariance. When the second measurement comes, ${z^{\prime }}_{1}^{i}$ can be obtained, then the filter based on measurement differencing can be used with the estimate of $x_{1}$ and its covariance as the a priori starting statistics.

### 3.3. Discussion

In this section, the stability and other properties of the proposed algorithm are discussed. To clearly show stability properties, the following assumptions are given

**Assumption** **4.** *The system matrix $F_{k}$ is invertible.*

**Assumption** **5.** *The system is globally observable, i.e., the pair $\left(F_{k},H_{k}\right)$ is observable, where $H_{k}=\left(H_{k}^{1},\ldots ,H_{k}^{N}\right)$.*

According to Theorem 4 in [19], under Assumptions 4 and 5, if we use the Kalman filter and the consensus method in Section 2.2, the estimation error is asymptotically bounded in mean square. It should be noted that the stability result relies only on the assumption of global observability, i.e., observability from the whole network and it can be further relaxed to global detectability. For the augmented state approach, the process noise and measurement noise are independent, then the stability analysis of the original state and measurement noise can be processed separately. In addition, the consensus is only processed on the original state. At this point, the stability of the estimation of the original state can be given by Theorem 4 in [19]. Since the system model remains unchanged, the measurement differencing approach has the similar stability property. The simulation results in the following section demonstrate that when the state is globally observable but not locally, the proposed methods can still work well.

The communication cost over the sensor network is also a big concern in the development of consensus algorithms. In the traditional consensus algorithms, only system state estimates are propagated between neighboring nodes. For the augmented state algorithm, since the strategy we adopt is to make only the system state estimation consistent, only the system state estimation propagates between neighbor nodes. For the measurement differencing algorithm, the state of the system does not change, and thus only the state of the propagation system. Therefore, compared with the traditional consensus algorithm, the two algorithms proposed in this paper do not increase the communication burden of the network system.

## 4. Simulations

A two-dimension tracking scenario is considered in this paper and the target moves in a horizontal plane. The target dynamic consists of the state $x=[p_{x},{\dot{p}}_{x},p_{y},{\dot{p}}_{y}{]}^{T}$, which can be modeled by (1) according to

(66) $$F_{k}=\left[\begin{array}{cccc} 1 & T & 0 & 0\\ 0 & 1 & 0 & 0\\ 0 & 0 & 1 & T\\ 0 & 0 & 0 & 1 \end{array}\right],Q_{k}=G_{k}\Delta G_{k}^{T}$$

where $\Delta =\mathrm{diag}(\left[w_{x}^{2},w_{y}^{2}\right])$, $w_{x}^{2}=w_{y}^{2}=1$, sample time *T* = 1 s and

(67) $$G_{k}={\left[\begin{array}{cccc} T^{2}/2 & T & 0 & 0\\ 0 & 0 & T^{2}/2 & T \end{array}\right]}^{T}$$

The target trajectory is generated by the above model with the following true initial state

(68) $$x_{0}={\left[2000\;m,10\;m/s,4000\;m,10\;m/s\right]}^{T}$$

In the simulations, initial states for filters are chosen randomly from $N(x_{0},P_{0})$ in each turn, where

(69) $$P_{0}=\mathrm{diag}\left(\left[50^{2}\;m^{2},5^{2}\;m^{2}/s^{2},50^{2}\;m^{2},5^{2}\;m^{2}/s^{2}\right]\right)$$

**Figure 2:**
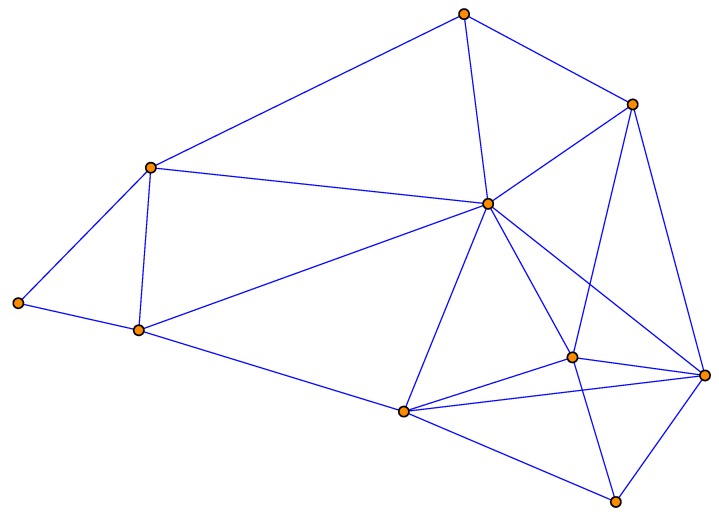
The topology of the sensor network used in the simulation.

There are 10 sensor nodes in the sensor network of which the graphical topology representation is shown in Figure 2. The measurement model is given by (2) and (5), where

(70) $$H_{k}^{i}=\left[\begin{array}{cccc} 1 & 0 & 0 & 0\\ 0 & 0 & 1 & 0 \end{array}\right]$$

(71) $$\Psi_{k}^{i}=\left[\begin{array}{cc} 0.5 & 0\\ 0 & 0.5 \end{array}\right]$$

The variance of measurement noise is $R_{k}^{i}=\mathrm{diag}\left(\left[{\left(20\;m\right)}^{2},{\left(20\;m\right)}^{2}\right]\right)$.

For performance comparison, the root mean-squared error (RMSE) of position is used as performance metric. In the target tracking application, the amount we focus on is position and velocity of target. The RMSE of position at time *k* is defined by

(72) $$RMSE_{p}\left(k\right)=\sqrt{\frac{1}{N}\sum_{n=1}^{N}\left({\left(p_{x,k}^{n}-{\hat{p}}_{x,k}^{n}\right)}^{2}+{\left(p_{y,k}^{n}-{\hat{p}}_{y,k}^{n}\right)}^{2}\right)}$$

where *N* denotes the number of Monte Carlo runs, $\left(p_{x,k}^{n},p_{y,k}^{n}\right)$ and $\left({\hat{p}}_{x,k}^{n},{\hat{p}}_{y,k}^{n}\right)$ are the true and estimated positions at the $n_{th}$ Monte Carlo run. The definition of RMSE of velocity is similar to the RMSE of position. The averaged RMSE (ARMSE) of position is defined by

(73) $$ARMSE_{p}=\sqrt{\frac{1}{N}\sum_{n=1}^{N}\frac{1}{K}\sum_{k=1}^{K}\left({\left(p_{x,k}^{n}-{\hat{p}}_{x,k}^{n}\right)}^{2}+{\left(p_{y,k}^{n}-{\hat{p}}_{y,k}^{n}\right)}^{2}\right)}$$

where *K* is the simulation step of a certain Monte Carlo. The definition of ARMSE of velocity is similar to the ARMSE of position.

We compare the performance of the traditional distributed consensus filter (DCF) in [19] which does not take the colored noise into consideration, the proposed distributed consensus filter for colored noise based on the augmented state approach (DCFCA) and the proposed distributed consensus filter for colored noise based on measurement differencing (DCFCD). The consensus step is L=5, the consensus weights of sensor nodes are set to $\pi^{i,j}=1/\left|N^{i}\right|$ if $j\in N^{i}$ and $\pi^{i,j}=0$ if $j\notin N^{i}$. As pointed by [19], only one consensus step per iteration is sufficient for stability. The more consensus steps can lead to more computational and communication burdens. However, the more consensus steps we use, the more accurate estimation we can obtain. The principle of selection of this variable is a compromise among the amount of calculation, communication burdens and the precision.

**Figure 3:**
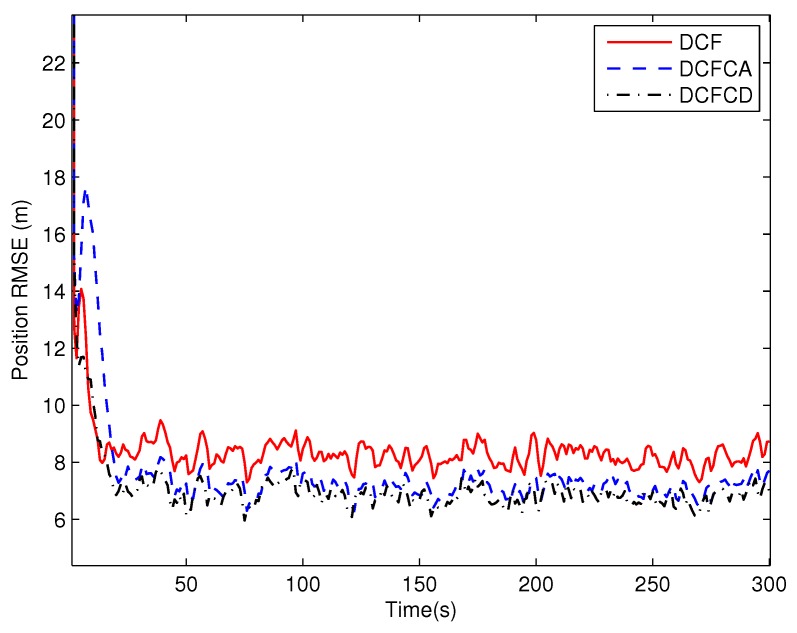
The position RMSE for different methods over time under colored noise.

**Figure 4:**
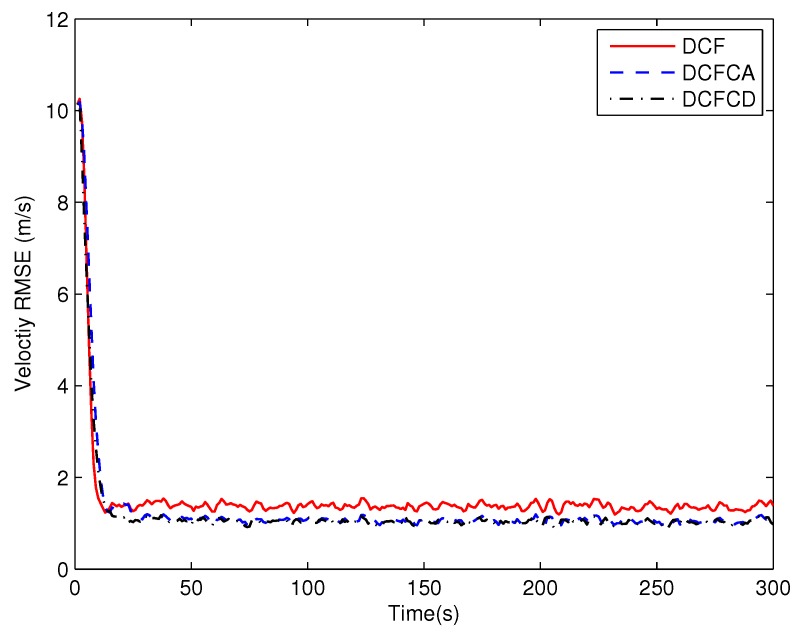
The velocity RMSE for different methods over time under colored noise.

Figure 3 plots the estimated position RMSE obtained by 100 Monte Carlo runs with 300 time steps for each Monte Carlo run. It can be seen from the figure that the position RMSE of DCFCD converges faster than the other two filters. The steady position RMSE of DCF is the largest of all. On the contrary, the steady position RMSE of DCFCD is the smallest of all. Besides, the steady position RMSE of DCFCA is a little larger than that of the DCFCD. The velocity RMSE obtained by 100 Monte Carlo runs are also given in Figure 4 and the result is similar to that of the position RMSE. It should be noted that the smaller RMSE indicates better performance. Therefore, we can see that performance of DCFCA and DCFCD is better than the traditional DCF. This is because that both of DCFCA and DCFCD take the colored noise into consideration while DCF dose not.

**Figure 5:**
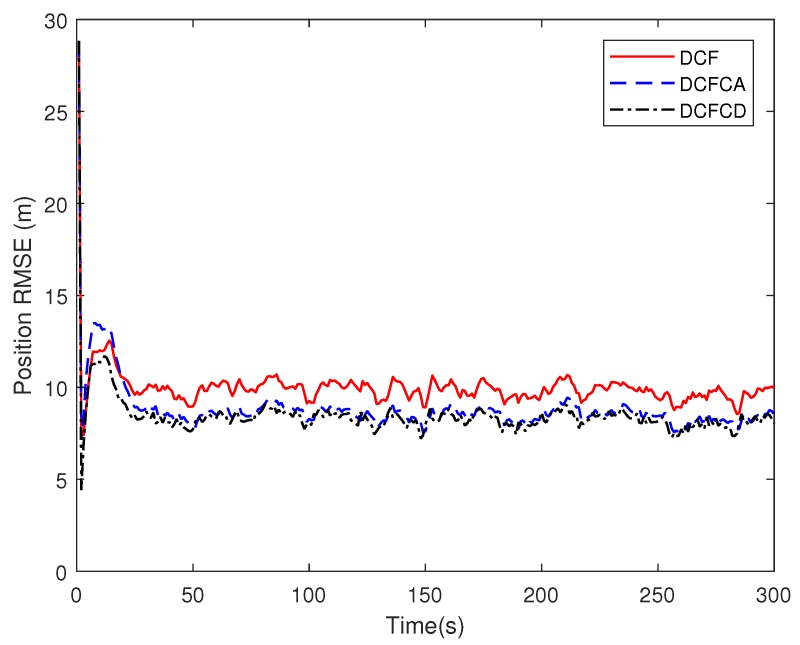
The position RMSE for different methods over time with global observability.

**Figure 6:**
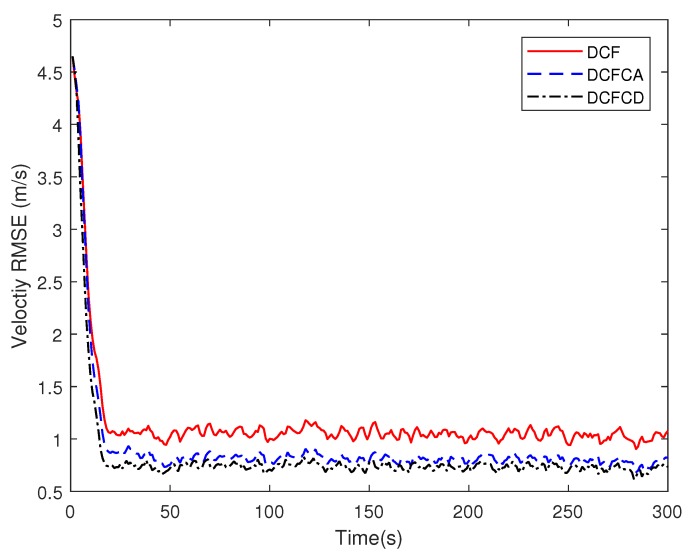
The velocity RMSE for different methods over time with global observability.

The state matrix (66) measurement matrix (70) guarantee that the system state is locally observable at each and every node in the network. To see performance of the three filters under the case where the state is globally observable but not locally, we make five of the ten sensors have the measurement matrix $H_{k}^{i}=\left[\begin{array}{cccc} 1 & 0 & 0 & 0 \end{array}\right]$ and the others have the measurement matrix $H_{k}^{i}=\left[\begin{array}{cccc} 0 & 0 & 1 & 0 \end{array}\right]$. The RMSE of the estimated position and velocity obtained by 100 Monte Carlo runs are shown in Figure 5 and Figure 6. It can be seen from the figures that all of the three filters work well under the case where the state is globally observable but not necessarily locally. We can also see that DCFCD has the best performance and DCF has the worst performance. In addition, all the filters have better performance under the case where the state is locally observable.

To evaluate performance of the three filters under different colored noises, we have $\Psi_{k}^{i}=diag\left(\psi ,\psi \right)$, where the scalar ψ indicates the correlation of the measurement noise. When the scalar ψ=0, the measurement noise becomes white. As ψ increases, the color of the measurement noise increases, which means that the measurement noise contains more low frequency components and less high frequency components. Table 1 and Table 2 given the estimated position ARMSE and velocity ARMSE obtained by 100 Monte Carlo runs with 300 time steps for each Monte Carlo run. It can be seen from the tables that as the ARMSE increase with the increase of the scalar ψ and the filters (DCFCA and DCFCD) that take the color of the measurement noise into account provide increasingly better performance compared to the traditional DCF method. As before, the DCFCD method has the best performance for different ψ.

The ARMSE of different measurement noise covariances are given in Table 3 and Table 4, if we have $R_{k}^{i}=diag\left(\delta^{2},\delta^{2}\right)$. It can be seen from the tables that the ARMSE increase with the increase of the δ and the filters ( DCFCA and DCFCD ) that take the color of the measurement noise into account show better performance compared to the traditional DCF method.

**Figure 7:**
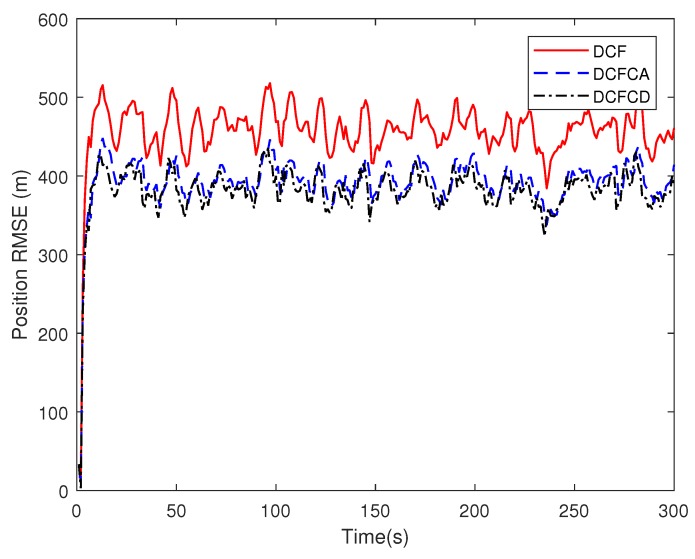
The position RMSE for different local filters of failure node without consensus.

**Figure 8:**
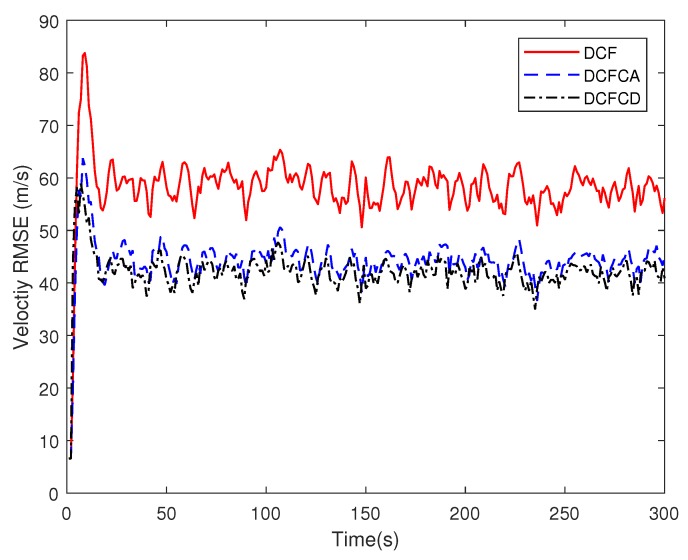
The velocity RMSE for different local filters of failure node without consensus.

**Figure 9:**
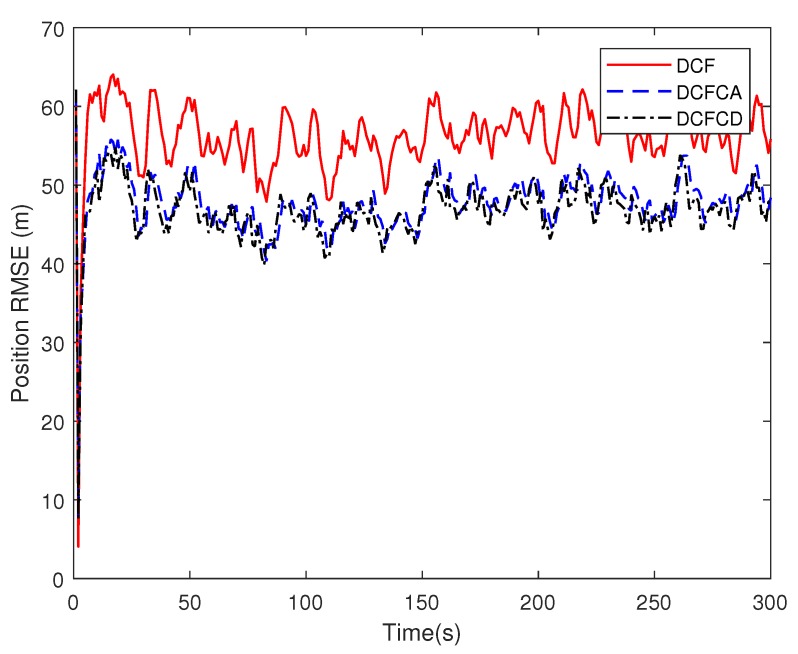
The position RMSE for different consensus filters of failure node.

**Figure 10:**
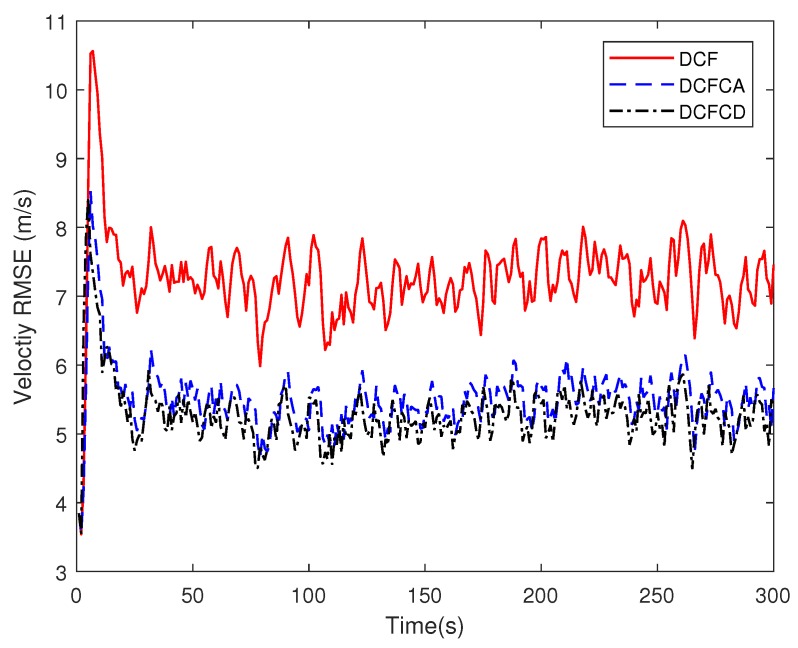
The velocity RMSE for different consensus filters of failure node.

When a node in the sensor network fails, the sensor noise may suddenly increase. At this point, if the local filter does not sense the fault, the node may be unstable. Here we assume that the noise standard deviation of certain node suddenly increases by 20 times due to the cause of the fault. The RMSE for local filter without consensus (consensus step L=0) of failure node are shown in Figure 7 and Figure 8. We can see that the RMSE are very large for different filters. If we make consensus step be L=5, the performance are much better, which can be seen in Figure 9 and Figure 10. This shows that the consensus filter has a certain degree of fault tolerance.

To evaluate performance of the three filters under white Gaussian noise, we have

(74) $$\Psi_{k}^{i}=\left[\begin{array}{cc} 0 & 0\\ 0 & 0 \end{array}\right]$$

**Figure 11:**
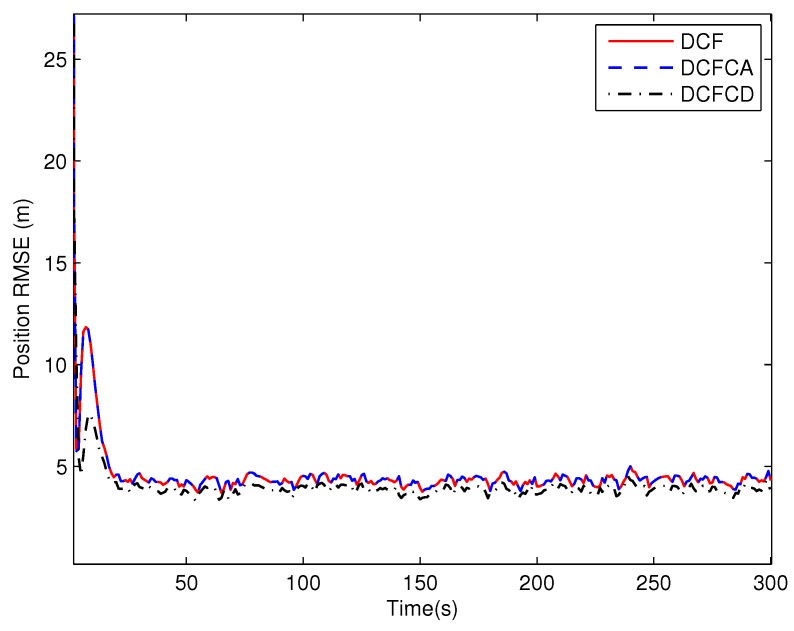
The position RMSE for different methods over time under white Gaussian noise.

**Figure 12:**
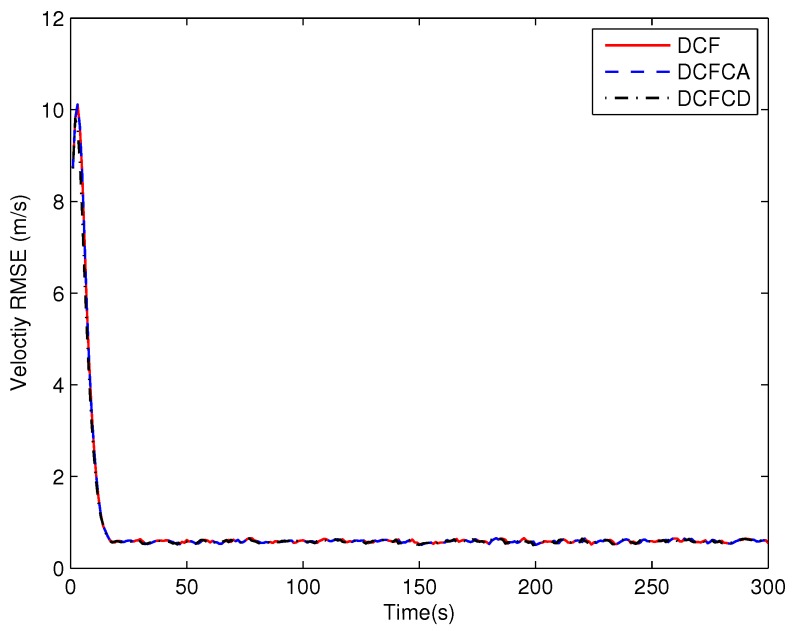
The velocity RMSE for different methods over time under white Gaussian noise.

The RMSE of the estimated position obtained by 100 Monte Carlo runs are shown in Figure 11. We can see from the figure that performance of the DCFCD outperform the DCF and the DCFCA while performance of DCF and DCFCA are almost the same. The reason is that the the DCF method is mathematically equivalent to the DCFCA method under white Gaussian measurement noise and the DCFCD method is actually a one step smoother for the estimation of the state. The RMSE of the estimated velocity obtained by 100 Monte Carlo runs are shown in Figure 12 and the result is similar to that of the position RMSE.

**Figure 13:**
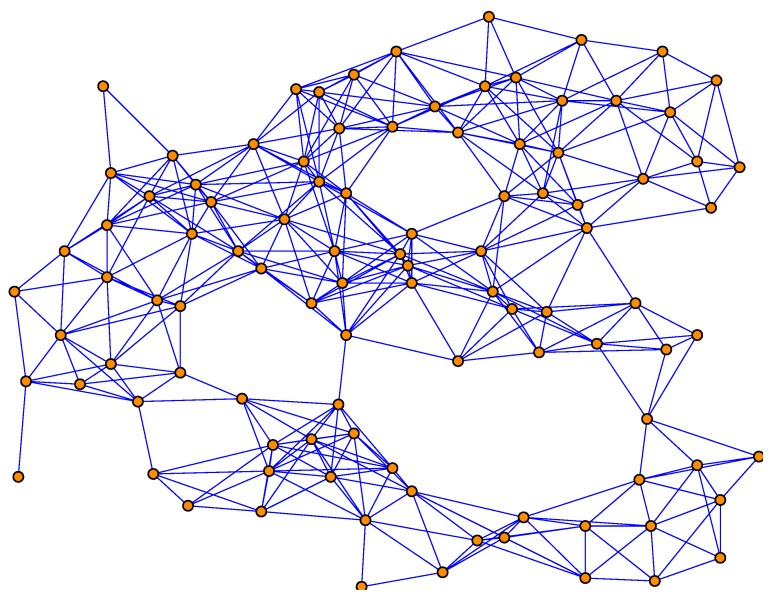
The topology of the sensor network with 100 sensor nodes.

**Figure 14:**
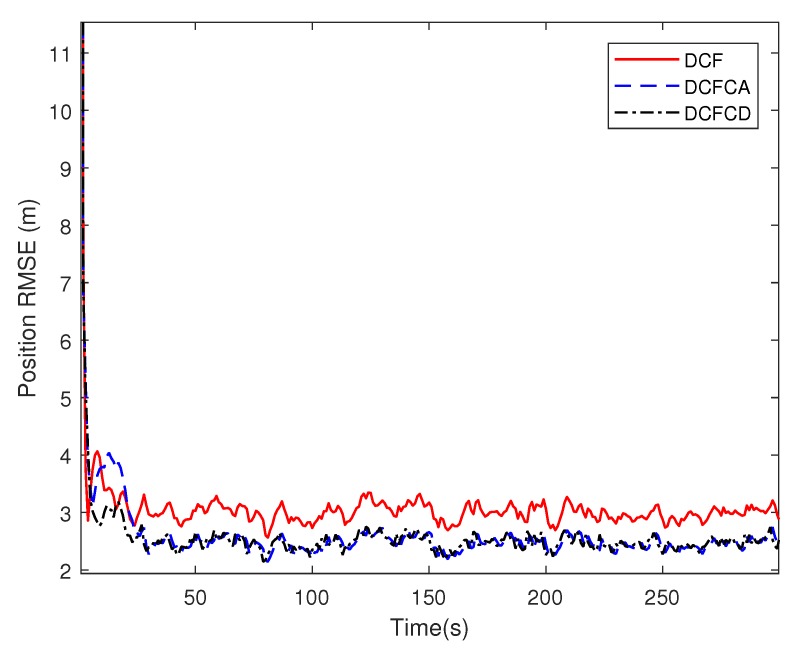
The position RMSE for different methods over time with 100 sensor nodes.

**Figure 15:**
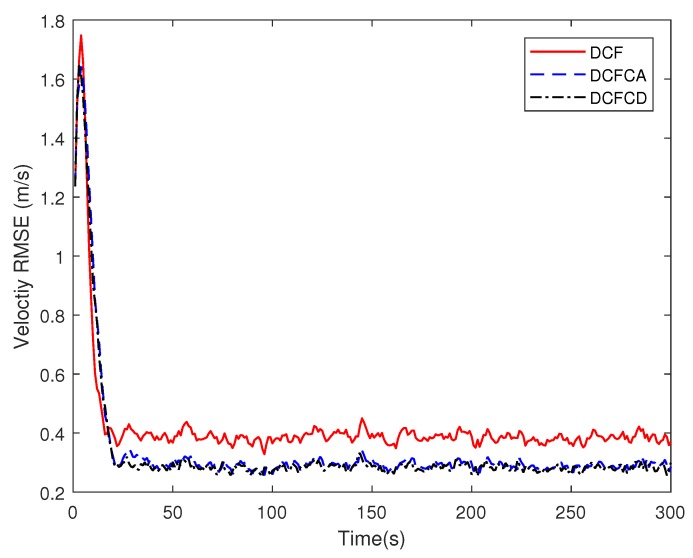
The velocity RMSE for different methods over time with 100 sensor nodes.

To show the performance of the proposed methods with large network size, we have performed a simulation with 100 sensors (see Figure 13) and the results are shown in Figure 14 and Figure 15. It can be seen from the figure that our proposed methods can still perform better than the traditional one in large network size.

## 5. Conclusions

This paper proposed the consensus-based distributed state estimate methods for distributed sensor network with colored measurement noise. The dynamics system model of each sensor node is modified to include the model of the colored measurement noise. Then the state is augmented to consist of the colored measurement noises. For each local filter, we only integrate the colored measurement noise of each local node into the augmented state to make the filter more effective. Moreover, the consensus is only carried out on original state without the colored measurement noise for each sensor node. To deal with the potential ill-conditioned computations and reduce the dimension of the augmented state approach, the consensus-based distributed filter based on measurement differencing scheme is proposed. The contribution of colored portion of the noise to the received signal is estimated in each sensor node, then this part is eliminated from the received signal. Simulation results show that the proposed methods outperform the traditional method, which does not take the colored measurement noise into account under the situation where the measurement noise is colored in the sensor network. Even for the white Gaussian noise, the performance of the proposed methods are not lower than the traditional method. The proposed algorithms are derived under assumption of linear system model and known statistic characteristics of colored measurement noise. In the future, we shall extend these algorithms to handling nonlinear models and unknown statistic characteristics of colored measurement noise.

## Figures and Tables

**Table 1:** Position ARMSE of different colored noises.

Methods	ψ=0	ψ=0.2	ψ=0.4	ψ=0.6	ψ=0.8
DCF	4.9628	5.4981	6.8682	9.2026	10.6130
DCFCA	4.9628	5.2320	6.4136	8.1365	9.4174
DCFCD	4.4384	4.8552	5.9054	7.6690	8.9543

**Table 2:** Velocity ARMSE of different colored noises.

Methods	ψ=0	ψ=0.2	ψ=0.4	ψ=0.6	ψ=0.8
DCF	0.8141	0.7640	1.0856	1.2654	1.4003
DCFCA	0.8141	0.7090	0.9712	1.0109	1.0312
DCFCD	0.7947	0.6905	0.9108	0.9288	0.9262

**Table 3:** Position ARMSE of different measurement noise covariances.

Methods	δ=5m	δ=10m	δ=15m	δ=20m	δ=25m
DCF	2.3499	4.5342	6.1405	7.9913	9.1410
DCFCA	2.1226	4.1622	5.5639	7.1183	7.9679
DCFCD	1.9234	3.8094	5.1714	6.7969	7.7378

**Table 4:** Velocity ARMSE of different measurement noise covariances.

Methods	δ=5m	δ=10mm	δ=15m	δ=20m	δ=25m
DCF	0.6127	0.8994	1.0441	1.0877	1.0977
DCFCA	0.5070	0.7686	0.8670	0.8854	0.8924
DCFCD	0.5029	0.7163	0.8075	0.8335	0.8512
